# A physically meaningful relationship between R50% and PTV surface area in lung SBRT

**DOI:** 10.1002/acm2.12964

**Published:** 2020-07-28

**Authors:** Dharmin D. Desai, Ivan L. Cordrey, E. L. Johnson

**Affiliations:** ^1^ Radiation Oncology CHI Memorial Hospital Chattanooga TN USA; ^2^ Department of Radiation Medicine University of Kentucky Chandler Medical Center Lexington KY USA

**Keywords:** lung, PTV mean dose, PTV surface area, R50%, R50% lower bound, SBRT

## Abstract

**Purpose:**

We propose a novel understanding of two characteristics of the planning target volume (PTV) that affect the intermediate‐dose spill in lung stereotactic body radiation therapy (SBRT) as measured by R50%. This phantom model research investigates two characteristics of the PTV that have a marked effect on the value of R50%: the mean dose deposited within the PTV (D_av_) and the surface area of the PTV (SA_PTV_).

**Methods:**

Using a phantom model provided by a CT of the IROC Thorax‐Lung Phantom® (IROC Houston QA Center, Houston, TX) and Eclipse® Treatment Planning System (Varian Medical Systems, Palo Alto, CA), we investigate the two characteristics for spherical and cylindrical PTVs. A total of 135 plans with tightly controlled PTV characteristics are employed. A lower bound for R50% (R50%min_∆r_) is derived and clearly establishes a relationship between R50% and SA_PTV_ that has not been fully appreciated previously.

**Results:**

The study of PTV D_av_ revealed a local minimum for R50% as a function of the PTV D_av_ at D_av_ ≈ 110% of Rx dose. As PTV D_av_ increases above this local minimum, R50% increases; while for PTV D_av_ less than this local minimum, the R50% value also increases. The study of PTV surface area (SA_PTV_) demonstrated that as the SA_PTV_ increases, the R50% increases if the PTV volume stays the same. The SA_PTV_ result is predicted by the theoretical investigation that yields the R50% lower bound, R50%min_∆r_.

**Conclusions:**

This research has identified two characteristics of the PTV that have a marked influence on R50%: PTV D_av_ and SA_PTV_. These characteristics have not been clearly articulated in the vast body of previous research in SBRT. These results could help explain plans that cannot meet the RTOG criteria for R50%. With further development, these concepts could be extended to provide additional guidance for creating acceptable SBRT plans.

## 1 INTRODUCTION

Stereotactic body radiation therapy (SBRT) was defined in 2014 as “a method of external beam radiotherapy (EBRT) that accurately delivers a high dose of irradiation in one or few treatment fractions to an extracranial target.”^1^ This technique of high‐precision, ultra‐hypofractionated EBRT is gaining widespread clinical use facilitated by convenient, commercially available equipment that makes SBRT practical even in community‐based radiation therapy facilities. Stereotactic body radiation therapy has been rapidly adopted as the standard of care for nonsurgical early‐stage lung cancer.^2^, ^3^, ^4^, ^5^, ^6^

The current clinical practice of SBRT for early‐stage nonsurgical lung cancer treatment is based on the clinical protocols provided by three RTOG studies: RTOG 0236,^7^ RTOG 0813,^8^ and RTOG 0915.^9^ A principle metric for plan acceptability is R50%. R50% is a derived unitless quantity obtained from the volume of the 50% prescription isodose cloud (IDC50%) and the planning target volume (PTV) volume as follows:

(1) $$R50\%\left(\mathrm{unitless}\right)=\frac{IDC50\%\mathrm{Volume}}{\mathrm{PTV}\mathrm{Volume}}$$

The IDC50% is a surrogate for damage to irradiated normal tissues as discussed in the work of Yang et al.^10^ The goal of SBRT treatment planning is to deliver a high dose to the target (for local tumor control) that meets or exceeds the minimum dose specified by the prescription while minimizing the IDC50% (to minimize normal tissue damage) and avoiding unacceptable dose to organs at risk (OAR).

The RTOG protocol^9^ provides guidance for acceptable values for R50% as function of the PTV volume measurement. Other RTOG‐defined quantities used to assess SBRT plan quality are D2cm^7^ (maximum Dose 2 cm from the PTV in any direction), V105%^9^ (105% prescription isodose volume outside of the PTV), and CI^9^ (Conformity Index = [100% prescription Isodose Volume]/[PTV Volume]). In addition, these protocols contain limits on various OARs, such as the spinal cord and ribs.

When considering the PTV dose heterogeneity there are three metrics available: ICRU HI,^9^ RTOG HI,^11^ and PTV mean dose (PTV D_av_). The ICRU HI, RTOG HI, and PTV D_av_ are defined, respectively, as follows:

(2) $$ICRUHI=\frac{\left(D_{2\%}‐D_{98\%}\right)}{D_{50\%}}$$

where D_x%_ = the minimum dose to x% of the PTV.

(3) $$RTOGHI=\frac{D_{max}}{D_{Rx}}$$

where D_max_ = “maximum dose in the PTV” and D_Rx_ = “prescription dose.”

And

(4) $$PTVD_{av}=meandoseinPTV$$

Many authors have reported investigations on Lung SBRT treatment plan quality by analyzing adherence to the various goals set forth in the RTOG protocols.^12^, ^13^, ^14^, ^15^, ^16^ Treatment planning outcomes have complex dependencies on many factors, both PTV and OAR related. However, we believe there are two important PTV‐related planning considerations that may have been underappreciated in this large body of work. Our analyses have indicated that both the PTV mean dose (PTV D_av_) and the PTV surface area (SA_PTV_) can offer important guidance in the treatment planning process. Therefore, the goal of this study is to develop an understanding of the behavior of R50% on these two PTV characteristics in a carefully controlled, systematic process that excludes complications resulting from a consideration of the full array of planning objectives. As such, any guidance derived from this study is intended to augment the planning process and not serve as a radical alteration. To achieve the study goals, we have utilized an anthropomorphic phantom model that allows a controlled environment to independently evaluate the effects PTV D_av_ and SA_PTV_ have on the plan metric R50%. We also derive a clinically useful and physically reasonable R50% lower bound that illuminates the dependence of R50% on SA_PTV_.

## 2 MATERIALS AND METHODS

This study utilized a treatment planning CT of the IROC Thorax‐Lung Phantom® (IROC Houston QA Center, Houston, TX) as the anthropomorphic phantom model. Complications from patient motion were not considered. We ignore the IROC PTV in the left lung and create a unique set of PTVs in the right lung with well‐controlled characteristics, eg, specific contour shape, dimensions, and overriding the electron density within the contour to create a uniform density of 0.9 g/cc. All treatment planning was performed on an Eclipse Radiation Treatment Planning System (RTPS) (Varian Medical Systems, Palo Alto, CA) using photon optimizer v15.6 with a final calculation via the AAA v15.6 algorithm on a 1 mm calculation grid size. Plans were created for delivery on a Varian TrueBeam STx® with 120 leaf HD MLC. None of the plans utilized in the study were subjected to standard QA procedures, but the RTPS and its model of the delivery system have been clinically validated by the IROC using the onsite planning and treatment delivery protocol for the Thorax‐Lung Phantom® and many direct patient QA measurements of clinical Intensity Modulated Radiation Therapy (IMRT), SBRT, and cranial SRS patient cases. All plans use Volumetric Modulated Arc Therapy (VMAT, RapidArc®) techniques. The arc geometry was limited to include only the PTV and right lung and used typical clinical arc limits and collimator angles.

All PTVs were created as simple geometric shapes of uniform physical density to simplify the problem and control as many variables as possible, thus isolating the PTV characteristics under study. The simple, well‐defined geometric shapes also allowed an analytic calculation of the PTV surface area.

**Fig. 1:**
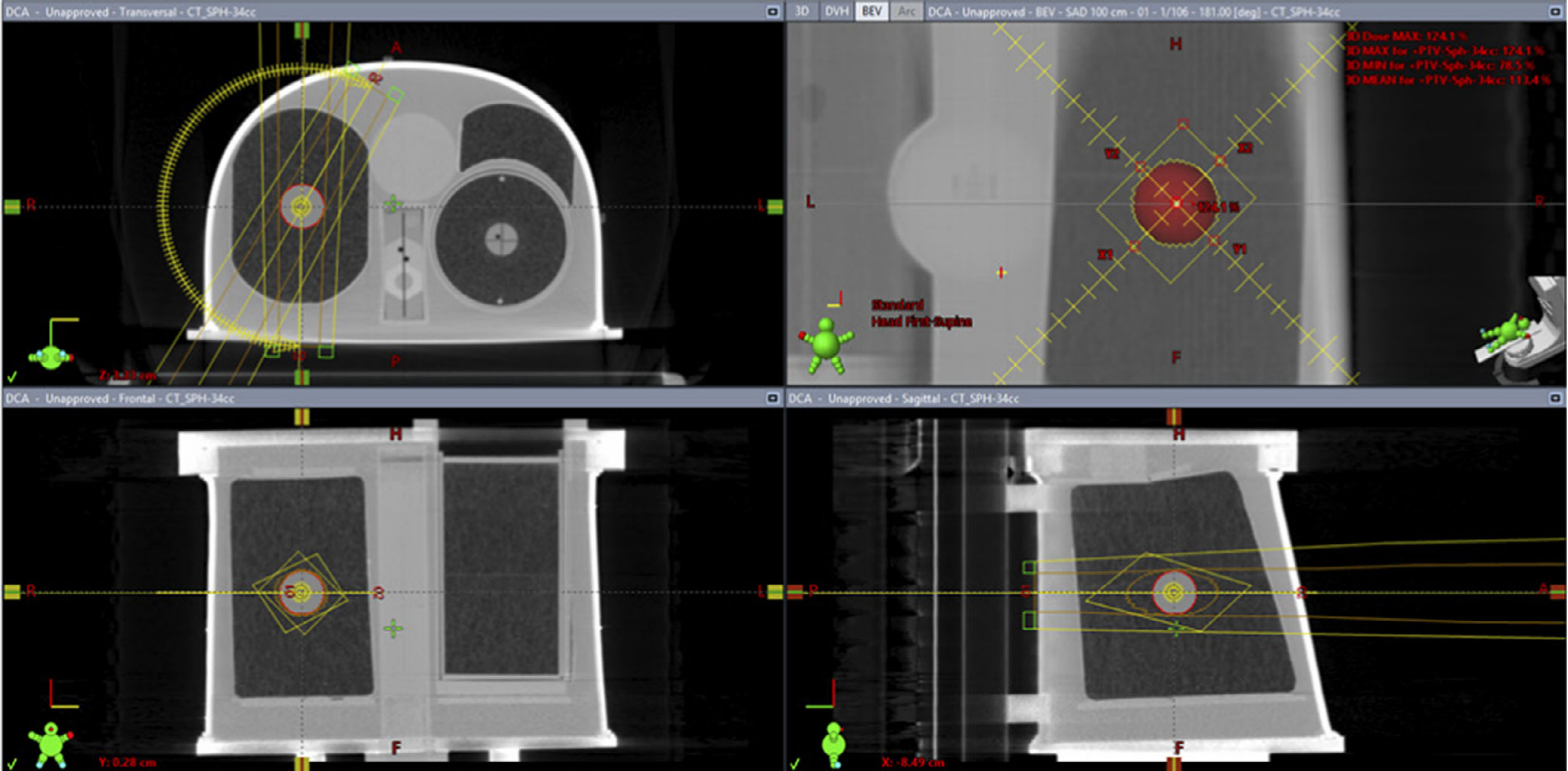
Geometry of the experimental plans run on the IROC Thorax‐Lung Phantom^®^. The PTV shown in the right lung is a density override volume and indicates the position of isocenter and the arc geometry used.

The geometric configuration of gantry, collimator, and phantom is shown in Fig. 1. All plans in this study are RapidArc 2‐arc VMAT plans having gantry angle limits between 30° and 181^o^ and collimator angles of 45^o^ or 330°.

All plans use a typical SBRT prescription (Rx) of 50 Gy in five fractions, and it was required that 95% of the PTV volume receive the Rx dose (ie, D95% condition).^17^, ^18^ The AAA algorithm was used in part because it is the most common clinical calculation algorithm in Eclipse. There is justification for this choice in an evaluation performed by Matsuoka et al.^19^ showing good precision for determination of the quantity V_50%_/V_100%_, a quantity similar to R50%. In addition, Matsuoka et al. showed that for SBRT targets with near uniform density, various dose indices such as D_95%_ and D_50%_ are essentially independent of dose calculation algorithm. Therefore, our phantom study with no respiratory motion and well‐defined target properties satisfies these conditions. All plans meet the requirements of RTOG protocols^7^, ^8^, ^9^ for D2cm, V105%, and CI.

### 2.A R50% dependence on PTV Dose Heterogeneity

To study the relationship between R50% and PTV dose heterogeneity, we generated four spherical volumes (9, 33, 53, and 81 cc) and four cylindrical shapes of the same volumes but varying elongation. We employed two different PTV shapes to control for PTV shape so the results for dose heterogeneity study can be clearly attributed to the dose heterogeneity independent of the PTV shape. All eight PTVs are located in the center of the right lung. The density of the PTVs was overridden to a typical 0.9 g/cc for all cases.

A series of ten plans for each PTV were created that control the PTV dose heterogeneity using only a PTV maximum dose constraint (PTV D_max_) and the prescription D95% condition. The PTV D_max_ constraint included an unlimited maximum dose and maximums ranging from 140% to 100% of the Rx dose decreasing in increments of 5%. This resulted in plans with progressively more dose homogeneity in the PTV. We use PTV D_max_ to contain the PTV dose heterogeneity within the limits of the prescription D95% and PTV D_max_. We examine these results in light of two more standard metrics for dose heterogeneity: ICRU HI and RTOG HI [defined by Eqs. (2) and (3), respectively]. We also study these results with a third metric PTV D_av_ defined by Eq. (4).

We report the results relative to PTV D_av_ because this is a reasonable measure of the actual global dose delivered to the target. D_av_ is also a readily available parameter in all commercially available RTPS and allows comparison with previous studies.

To further understand the dose distribution, we examined the average dose in the shell of the IDC50% that is outside the PTV. For notational simplicity, we define IDC50%shell as that part of IDC50% that is not part of the PTV.

(5) IDC50%shell=IDC50%‐PTV

This evaluation of PTV dose heterogeneity generated a total of 80 plans (4 volumes × 2 shapes × 10 D_max_ constraints).

### 2.B Dependence on PTV surface area

To study the relationship between R50% and SA_PTV_, we generated a large set of PTVs of both spherical and cylindrical shape and having varying surface area. All PTVs were defined to have a uniform density of 0.9 g/cc. The optimizer PTV D_max_ constraint was held constant at 120% of prescription yielding a PTV D_av_ of approximately 110% of Rx.

With reference to RTOG 0915,^9^ we generated 11 spherical PTVs ranging in volume from 2 to 163 cc denoted as “Sphere” (or “Sph”). A set of cylindrical PTVs were generated having volumes equal to the 11 spherical PTVs and a maximum length equivalent to the maximum dimension given in RTOG 0236 thus fixing diameter for a given maximum length. These cylinders were placed in 2 distinct orientations: cylindrical axis in the superior/inferior direction (denoted as “RTOG Horizontal Cylinders” or “RTOG Horz Cyl”); and a second set with the cylindrical axis in the anterior/posterior direction (denoted as “RTOG Vertical Cylinders” or “RTOG Vert Cyl”). To provide additional variation in the surface area, we created an additional set of cylindrical PTVs with the same volume as the spherical PTVs but very long cylindrical axes and thus smaller radial dimensions than the cylindrical PTVs mentioned above. The maximum cylinder height was capped at 8 cm, however, to avoid clinical irrelevance. These new cylinders are also oriented in both the “horizontal” and “vertical” directions previously described. The dual cylinder orientations were used to more directly test the PTV surface area dependence of R50%, by controling for PTV orientation relative to the delivery geometry of the beams. This section of the study generated a total of 55 PTVs and associated plans.

### 2.C R50% Limit Derivation

**Fig. 2:**
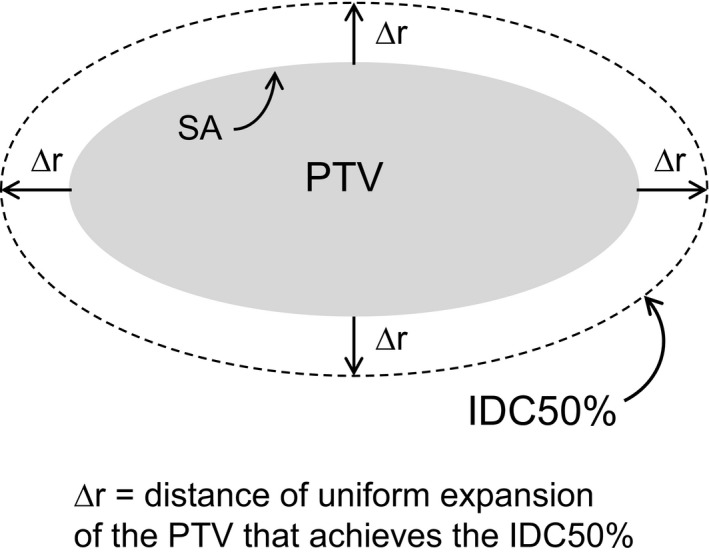
An arbitrary shape planning target volume of surface area SA is expanded by ∆r to achieve the IDC50% volume.

To derive the R50% limit in highly conformal treatment like SBRT, begin by considering Fig. 2.

The volume of the IDC50% can be determined by a differential shell expansion of the PTV. If the expansion is vanishingly small, ∆r becomes dr. In this differential shell, the volume is given by

(6) $$VolumeofDifferentialShell_{1}=V_{1}=SA_{PTV}\times dr$$

This is strictly correct only if the differential shell is truly an infinitesimal expansion. This expansion generates a new volume and that volume, V_1_, has a surface area, SA_1_. Now that volume, V_1_, can be expanded by another dr into volume V_2._

(7) $$V_{2}=SA_{1}\times dr$$

V_2_ has a surface area SA_2_, and

(8) $$SA_{2}>SA_{1}$$

This process continues through the entire infinitesimal differential shell series of nested shells such that

(9) $$SA_{\left(i+1\right)}>SA_{i}$$

Thus, SA is a function of r, SA(r), where we are concerned with r in the range from the PTV surface outward. For notational simplicity, adopt the notation that r = 0 at the surface of the PTV, and r = ∆r at the surface of IDC50%.

Continue adding differential shells until the surface reaches IDC50% (at distance ∆r). Thus, strictly speaking,

(10) $$V_{IDC50\%}=V_{PTV}+\int_{0}^{\Delta r}SA\left(r\right)dr$$

Since each differential shell surface area is larger than the one nested inside it, one can see that

(11) $$V_{IDC50\%}>V_{PTV}+SA_{PTV}\times \Delta r$$

Divide both sides of the inequality by V_PTV_ to obtain the following:

(12) $$R50\%>1+\frac{SA_{\mathrm{PTV}}}{V_{\mathrm{PTV}}}\times \Delta r$$

We can now define a lower bound for R50%. This lower limit we name R50%min_∆r_.

(13) $$R50\%min_{\Delta r}=1+\frac{SA_{\mathrm{PTV}}}{V_{\mathrm{PTV}}}\times \Delta r_{\min}$$

Values of $\Delta r_{\min}$ are difficult to determine analytically, but estimates can be obtained from previous lung SBRT treatment planning studies. For example, Hoffman et al.^20^ determined lung SBRT Gradient Measure (GM) values obtained from a multicenter analysis of 317 lung SBRT plans. They define GM as the difference, in centimeters, of the equivalent sphere radii of the 50% and 100% isodose volumes ($R_{\mathrm{eq}}^{50\%}$ and $R_{\mathrm{eq}}^{100\%}$, respectively). Thus,

(14) $$GM=R_{\mathrm{eq}}^{50\%}‐R_{\mathrm{eq}}^{100\%}$$

By comparison, for a perfectly conformal plan with Conformality Index = 1, the volume of the 100% isodose volume is identical to and spatially coincident with the PTV volume. Thus, for the equivalent sphere, $R_{\mathrm{eq}}^{100\%}$ = radius of the equivalent sphere PTV. In such case, the GM is equivalent to the ∆r as defined in Fig. 2. Thus, the GM values from Hoffman et al can be used as approximations to our ∆r_min_. The GM values obtained from Hoffman, et al. are binned by PTV Volume and range from 0.84 to 2.13 cm for 1.8–3.8 cc to >163 cc, respectively. So, Eq. (13) becomes:

(15) $$R50\%min_{\mathrm{GM}}=1+\frac{SA_{\mathrm{PTV}}}{V_{\mathrm{PTV}}}\times GM$$

where the subscript GM designates ∆r_min_ = GM from Hoffman et al. (Ref. [20], Table 1]).

In principle, the limit in Eq. (13) should hold true even if the value of ∆r is refined for different clinical presentation and different densities in the IDC50%shell, such as SBRT for liver, spine, or even cranial SRS.

## 3 RESULTS

### 3.A R50% dependence on PTV D_av_

**Fig. 3:**
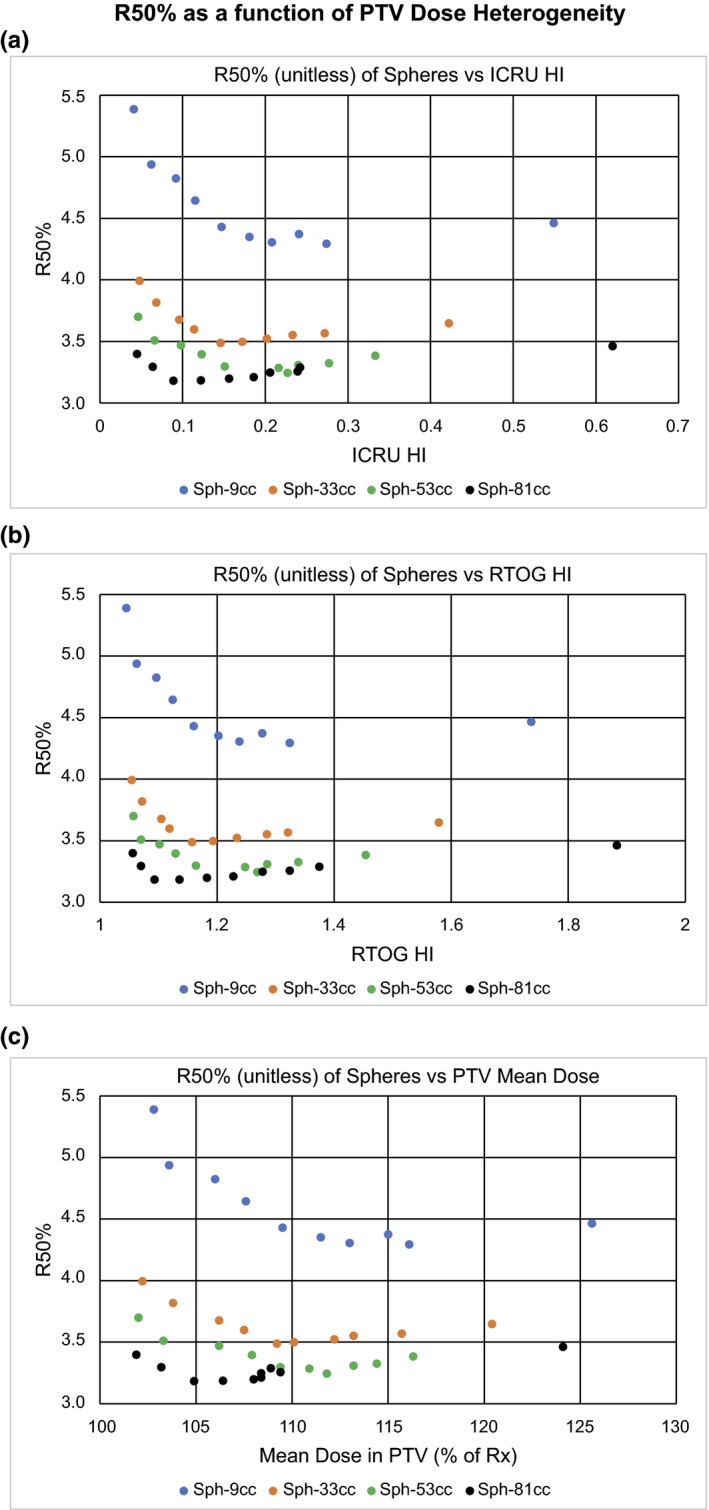
R50% as a function of planning target volume (PTV) dose heterogeneity. Plots of R50% as a function of various measures of PTV dose heterogeneity. (a) R50% vs ICRU HI. (b) R50% vs RTOG HI. (c) R50% vs PTV Mean Dose (PTV D_av_) expressed as a percentage of the prescription dose.

**Fig. 4:**
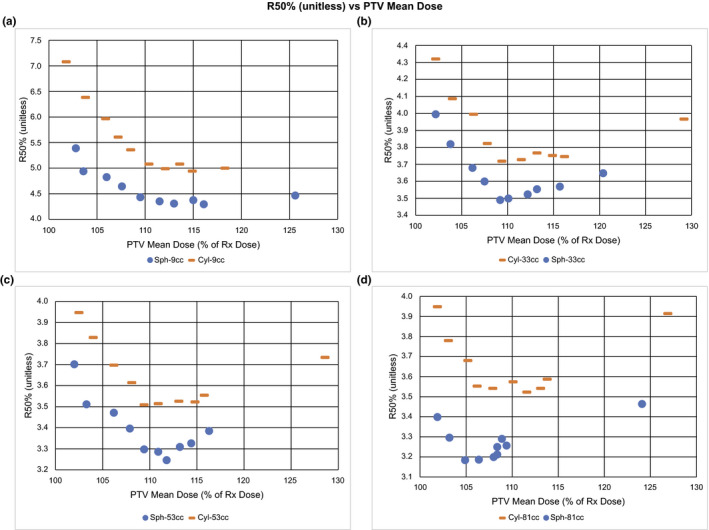
R50% (unitless) vs planning target volume (PTV) mean dose. Plots of R50%(unitless) vs PTV Mean Dose (D_av_) as % of Rx Dose for a tissue equivalent PTV density (0.9 g/cc) of both spherical and cylindrical shapes for volumes: (a) 9 cc, (b) 33 cc, (c) 53 cc, and (d) 81 cc. Note the minimum in R50% that occurs near D_av_ = 110% of Rx dose for all volumes and shapes.

The PTV dose heterogeneity can be described in many ways. Figure 3 shows three different descriptions of PTV dose heterogeneity: ICRU HI, RTOG HI, and PTV D_av_ expressed as a percentage of the prescription dose. The PTV shape (sphere or cylinder) has very little effect on the overall trend, thus, the trend is attributable to the dose heterogeneity alone. It is clear from these plots that each measure of heterogeneity contains very similar information. PTV D_av_ is reported directly by all commercially available RTPS and is easy to conceptualize. In addition, D_av_ is directly correlated with tumor control.^17^ Therefore, we choose PTV D_av_ as our metric of choice for the evaluation of PTV dose heterogeneity. Results for the dependence of R50% on PTV D_av_ are summarized in Fig. 4.

The three regions of interest seen in all four panels of Fig. 4 are PTV D_av_ < 105% Rx, PTV D_av_ > 115% Rx, and the interval between 105% and 115% Rx. There appears to be a relatively broad local minimum in R50% in the interval between 105% and 115% with increases outside this range. The R50% local minimum region as a function of PTV D_av_ (between 105% and 115% of the Rx dose) appears to vary slightly with PTV volume. The R50% local minimum as a function of PTV D_av_ occurs at D_av_ ≈ 110% Rx.

**Fig. 5:**
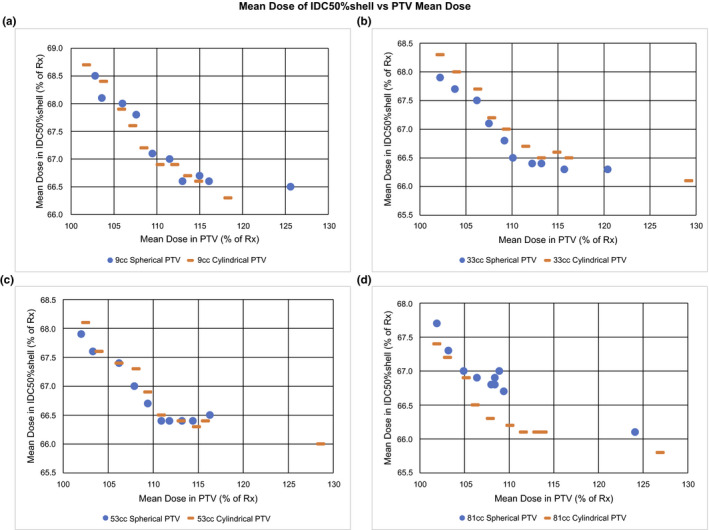
Mean dose of IDC50%shell vs planning target volume (PTV) mean dose. Plots of IDC50%shell Mean Dose vs PTV Mean Dose (D_av_) as percent of prescription Dose (% of Rx) for a tissue equivalent PTV (0.9 g/cc) of both spherical and cylindrical shapes for volumes: (a) 9 cc, (b) 33 cc, (c) 53 cc, and (d) 81 cc. Note the marked increase in IDC50%shell Mean Dose as the PTV D_av_ approaches the Rx dose.

We also examined the mean dose in the IDC50%shell. Figure 5 shows that as PTV D_av_ decreases below the local minimum (D_av_ ≈ 110%), the mean dose in the IDC50%shell increases for all PTV volumes, ie, the dose spill outside the PTV increases as the PTV dose becomes more homogeneous.

### 3.B Dependence on PTV surface area

**Fig. 6:**
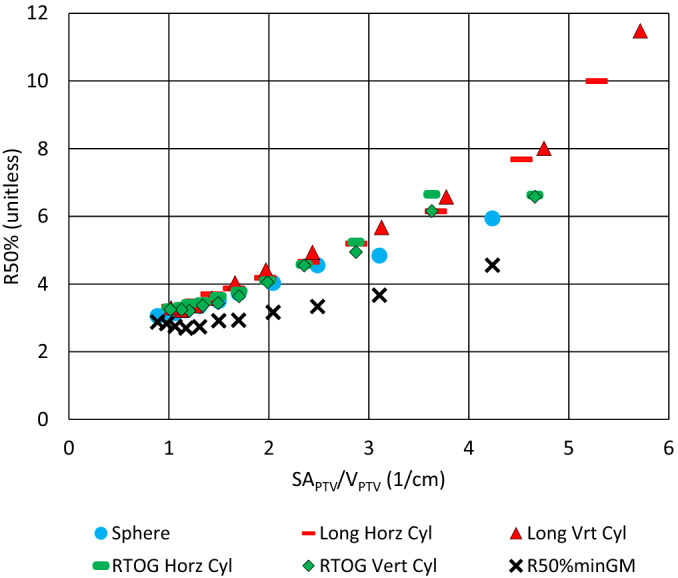
The graph R50% vs SA_PTV_/V_PTV_ showing all 55 plans of the Surface Area study. Also shown is R50%min_GM_ [Eq. (15)] using the GM values from Hoffman et al.^20^ Notice that the lower bound R50%min_GM_ is always <R50% for all plans and thus is indeed a lower bound.

**Fig. 7:**
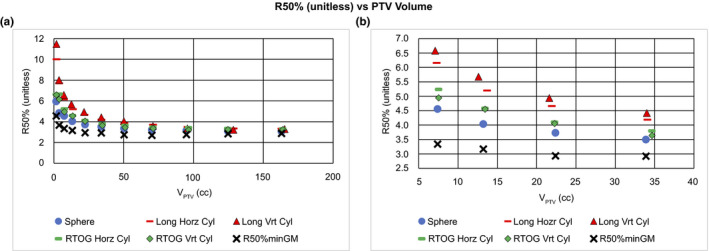
R50% (unitless) vs planning target volume (PTV) volume. The standard graph of R50% vs V_PTV_ for all 55 plans of the Surface Area study. Also graphed is R50%min_GM_ [Eq. (15)] obtained using the GM values from Hoffman et al.^20^ (a) The full dataset. Notice that the lower bound R50%min_GM_ is always less than R50% for all plans and thus is indeed a lower bound. (b) Is a highly magnified version of the same plot to demonstrate the vertical stacking of the R50% values for nearly the same volume. This vertical stacking corresponds to the Surface Area (SA) of the individual PTVs with some cylinder orientation evident as a perturbation on the larger surface area effect.

The results of the PTV surface area study are summarized in Figs. 6 and 7. Figure 6 shows R50% for all 55 plans plotted as a function of the SA_PTV_/V_PTV_ for all volumes and PTV shapes (spheres and various cylinders). Also displayed is the numerical value of R50%min_GM_ [Eq. (15)], which is the limit R50%min_∆r_ that uses ∆r_min_ = GM values given by Hoffman, et al.^20^ Notice that the lower bound R50%min_GM_ is always less than the planned R50% and thus is indeed a lower bound.

Figure 7 provides the same data as Fig. 6 but alternatively plotted as a function of PTV volume. As seen in Fig. 7(a), R50% rises sharply as the PTV Volume approaches zero. Again, it is evident that the R50%min_GM_ appears to be a true lower bound for R50% regardless of PTV volume. Figure 7(b) is a magnified view of a limited range of the data in 7(a). Here, one can see a vertical stacking of the R50% values that depends on the SA_PTV_ for the same nominal V_PTV_. R50% and SA_PTV_ for various PTV shapes and orientations are summarized in Table 1 for a nominal V_PTV_ = 34 cc. The dependence of R50% on SA_PTV_ is evident and consistent with the trend seen for the 34 cc volume in Fig. 7(b). That is, the largest SA_PTV_ corresponds to the largest R50% value. For a given cylindrical SA_PTV_ value, the orientation of the cylinder axis does play some role in the plan R50% obtained. It should be noted that the actual volume of the PTVs may not be exactly 34 cc due to voxel discretization limitations in the RTPS.

**Table 1:** Results from Surface Area study for planning target volumes (PTVs) having a nominal volume of 34 cc. Notice the association with the stack of points at about 34 cc as shown in Fig. 6(b). There is also a second‐order effect of cylinder orientation on R50%. Note that a larger SA_PTV_ correlates with a larger R50% for the same volume.

PTV description	SA_PTV_	R50%	Actual V_PTV_
Long Vrt Cyl	67.0	4.42	34.1
Long Horz Cyl	67.0	4.18	34.1
RTOG Horz Cyl	59.0	3.79	34.7
RTOG Vrt Cyl	59.0	3.64	34.7
Sphere	50.8	3.50	33.9

## 4 DISCUSSION

### 4.A R50% dependence on PTV D_av_

The observed R50% local minimum in PTV D_av_ seen in Fig. 4 can also be seen in the results of previous work.^12^, ^21^ However, these and other studies made little comment about the phenomenon. This R50% local minimum in PTV D_av_ is an interesting result of this study. The R50% local minimum region as a function of PTV D_av_ occurs between 105% and 115% of the Rx dose and appears to vary slightly with PTV volume. We are not certain that this minimum behavior will hold when other treatment planning constraints are applied, but it is a possible characteristic in a general treatment planning scenario.

The R50% local minimum as a function of PTV D_av_ as shown in Fig. 4, implies that there are two processes at work. One process dominates at the high end where PTV D_av_ is large while a different process dominates when PTV D_av_ is low. Where PTV D_av_ is large, the general result that R50% is increased seems reasonable given that more photon energy fluence is required to deliver a higher PTV mean dose. This higher photon energy fluence results in larger entrance doses around the PTV likely contributing to the larger R50%. On the low PTV D_av_ end, the rise in R50% as the PTV dose homogeneity increases may be explained by the photon energy fluence distribution. Because these are VMAT RapidArc plans, to achieve a more homogeneous PTV dose while maintaining the D95% prescription, the optimizer must force dose out of the PTV center and into the PTV periphery. This allows for the dose to the PTV center to remain close to the prescription dose while increasing the dose in the periphery to a level consistent with prescription dose. The PTV is effectively very thin at the periphery and so there is less PTV material to absorb the beam. This would increase the exit fluence from the PTV periphery. The higher exit fluence would contribute to a larger R50% and, thus, more dose would be deposited in the IDC50%shell. This effect is illustrated in Fig. 5 where the dose in the IDC50%shell is seen to increase as the dose in the PTV becomes more homogeneous as indicated by a reduction in the PTV D_av_. We acknowledge that there is little clinical justification for seeking a homogeneous PTV dose in SBRT. One could even argue that this is contrary to the standard clinical practice of SBRT. Previous recommendations^5^ have encouraged doses as high as 130% of Rx in the PTV. The point of this study, however, is to understand the effect that PTV dose heterogeneity, quantified by PTV D_av_, has on R50%. Many previous studies have shown how a heterogeneous dose within the PTV is desirable for a steep dose falloff surrounding PTV.^13^, ^14^, ^15^, ^16^, ^22^, ^23^ Our study clearly shows that this forced dose heterogeneity may only be useful to a point, that being the R50% local minimum in PTV D_av_. Beyond this point, increasing the PTV dose heterogeneity will increase the R50%. Many of the steep dose falloff studies are for cranial radiosurgery targets.^14^, ^15^, ^16^, ^22^, ^23^ Most of the radiosurgery target volumes are <10 cc and many involve SRS cones or a GammaKnife delivery platform. It is conceivable that the exact location of the R50% local minimum in PTV D_av_ could be optimizer and delivery technique dependent.

It should be noted that the difference between the spherical and cylindrical PTVs in Fig. 4 shows roughly the same local minimum in R50%. This implies that the local minimum observed is consistent regardless of PTV shape. The differences between the spherical and cylindrical PTVs are exposed in the study of PTV surface area.

The immediate clinical effect of this study is to alert the treatment planner that both homogenous PTV doses and extreme heterogeneous PTV dose distributions are undesirable if the goal is to obtain optimal values of R50%. Dosimetrists, Physicists, and Physicians need to be aware of this phenomenon.

### 4.B Dependence on PTV surface area

Dependence of R50% on SA_PTV_ appears to be underreported in the literature. The results of the PTV surface area study are illustrated in Fig. 6. R50% is seen to be highly correlated with SA_PTV_/V_PTV_ and very nearly linear regardless of PTV shape. One would expect this result from the R50%min_∆r_ expression given by $R50\%min_{\Delta r}=1+\frac{SA_{\mathrm{PTV}}}{V_{\mathrm{PTV}}}\times \Delta r_{\min}$ [Eq. (13)]. This clearly establishes the relationship of R50% to both volume of the PTV and the surface area of the PTV. However, there is also a slight curvature to the distribution owing to the PTV Volume dependence of ∆r = GM that was observed in the clinical data of Hoffman et al.^20^ This ∆r dependence on PTV Volume is also visible in the R50%min_GM_ lower bound.

The spherical PTVs always have the smallest R50% of any given volume. A sphere also has the smallest surface area to volume ratio of any three‐dimensional geometric object of arbitrary shape. Again, this supports the correlation of SA_PTV_ to R50% that one would expect from Eq. (13).

The data in Fig. 6 are clustered for small values of SA_PTV_/V_PTV_ in part because, for the larger volume geometric figures, the surface area to volume ratio naturally decreases and shape becomes less important. Furthermore, because of limitations placed on the PTVs when they were created, the cylinders all converge toward having a diameter ≈ height, which is the minimum surface area configuration of a cylinder. The larger values of SA_PTV_/V_PTV_ correlate with the smaller volume PTVs. For small 3D volumes, the SA_PTV_/V_PTV_ ratio becomes higher and diverges more for spheres and cylinders. These aspects of the data are also predicted by Eq. (13).

The gradient metric (GM) differs slightly from the ∆r of R50%min_∆r_ in that GM is calculated using effective spheres, not the actual shape of the PTV. Thus, GM will correlate better with ∆r for a spherical PTV than for a cylindrical PTV. The actual ∆r is likely dependent on the delivery geometry, so different treatment machines probably have slightly different ∆r values. This hypothesis would predict that a highly noncoplanar delivery from a CyberKnife (Accuray, Sunnyvale, CA) would have a smaller ∆r because the treatment delivery geometry could be optimized to the specific PTV shape.

One can investigate the limits of R50%min_∆r_ by examining the extremes of ∆r = r_50_ − r_100%_. If ∆r = 0 (indicating an infinitely steep, physically impossible dose drop‐off), one sees that in Eq. (13), R50%min_∆r_ = 1 indicating that the IDC50% = IDC100%. Clearly this is the absolute unphysical limit of R50% when IDC50% = PTV Volume. Furthermore, one sees that if ∆r is very large, the R50%min_∆r_ would be governed more strongly by SA_PTV_/V_PTV_. This is more likely to be true for lung SBRT, where the IDC50%shell is low‐density lung with a low electron stopping power, than for cranial SRS or liver SBRT where the IDC50%shell is soft tissue, and thus higher electron stopping power.

It is also likely that ∆r is dependent on the average aperture size as determined by the optimizer. For larger PTVs, the average aperture size would typically be larger and thus we would predict that ∆r is larger for large PTVs. The converse would be expected true for small PTVs. Of course, this is the result as reported by Hoffman et al.^20^ through their GM values.

The relationship R50% > 1 + SA_PTV_/V_PTV_ × ∆r also suggests that when assessing two PTVs of the same volume, the PTV with the larger surface area will have larger R50%. This accounts, in part, for the vertical spread of the R50% data in Fig. 7 that appear as a vertical stack of different plan data points of same/similar volume and is demonstrated in Table 1 for one case (V_PTV_ ≈ 34 cc).

We also note similarity of the PTV surface area study results to the recent publication of Goldbaum et al.^25^ working in cranial SRS. In that work, the authors hypothesized that “an increase in TV12 [intermediate dose spill] could be related to an increase in the surface area of the target, and the surface area of the target could be quantified by analogy with an ellipsoid.”^25^ Their attempt to use effective ellipsoids to quantify the PTV surface area did not prove productive. However, as we see from this work, the PTV surface area is a decisive factor in predicting the R50% limit given by R50%min_∆r_.

The results shown in Fig. 7(a) are markedly similar to clinical results published previously: Ref [26, Fig. 3], Ref [20, Fig. 3], Ref [24, Fig. 4], and Ref [11, Fig. 1(b)]. These other publications make no specific comment about the origin of this overall trend. This work, however, provides a clinically useful and physically reasonable explanation of the trend in R50% as a function of V_PTV_. The smaller surface area PTV (sphere) always has a smaller R50% for any given PTV volume. Furthermore, this general PTV surface area trend is maintained even when cylinders are oriented differently as shown in Table 1. Thus, the PTV orientation is only a minor perturbation on the overall PTV surface area effect.

Considering what the PTV surface area represents, it should not be surprising that PTV surface area has a marked effect on R50% because the PTV surface area is a direct measure of how much nontarget tissue is exposed to the high dose of the PTV: PTV surface area is the interface between target and nontarget tissue. Given the same degree of conformality, a larger PTV surface area in a lung SBRT plan means more lung tissue is exposed to the highest dose region. That exposure would propagate out to all isodose lines outside the target including the 50% isodose line defining the R50%.

Since PTV surface area markedly affects R50%, it would be advantageous to know this value in all clinical situations. At present we are aware of no commercially available RTPS that can report the surface area of a segmented structure. This may be why the importance of surface area has not been previously recognized. Having this statistic available in the treatment planning systems would make the study of PTV surface area possible in clinical studies.

Other authors have observed PTV shape dependence^27^ and have noted that a concave PTV has a higher R50% than a comparable volume fully convex PTV. Naturally, a concave PTV will have a higher surface area than a convex PTV of same volume. PTV surface area may prove a useful metric in future studies involving PTV shape.

The immediate clinical impact of this PTV surface area study is the understanding that for PTV shapes that are distinctly nonspheroidal, the R50% will be larger for the same volume and thus it will be more difficult to meet the R50% criterion given in the RTOG protocols^7^, ^8^, ^9^ for such plans. The derived R50%min_GM_ [Eq. (15)] is clinically useful in its own right. But it also clearly establishes the relationship of R50% with the PTV surface area (SA_PTV_).

The two PTV characteristics, PTV D_av_ and SA_PTV_, have a marked impact on the value of R50% and can be summarized as follows:

PTV Mean Dose (D_av_): Heterogeneity in the PTV dose distribution, as indicated by PTV D_av_, affects the value of R50%. There is a local minimum in R50% for PTV D_av_ ≈ 110% prescription dose.

PTV Surface Area: As the PTV surface area increases, the R50% naturally increases if the PTV volume stays the same.

The principle goal of this work is to illuminate the effects of the PTV characteristics, PTV D_av_, and SA_PTV_, on the plan metric R50% and to develop a conceptual understanding of these effects. This will allow future investigations to be more detailed and discuss how the entanglement of other characteristics is affecting the in‐depth study of a single PTV characteristic.

## 5 CONCLUSIONS

This research has identified two critical characteristics of the PTV that have not been clearly articulated in the vast body of previous research in lung SBRT: PTV D_av_ and SA_PTV_. Equation (13) clearly establishes a relationship between R50% and SA_PTV_ that has not been fully appreciated previously. This research provides new insight on a large body of previously published work related to lung SBRT. A better understanding of the relationship of these PTV characteristics to the ultimate R50% could provide improved guidance for determining when an optimal SBRT plan is achieved. Future work will provide more detailed descriptions of how these PTV characteristics affect R50%.

## CONFLICT OF INTEREST

None of the authors of this research have conflicts of interest to disclose.
